# Microbiological Air Quality and Drug Resistance in Airborne Bacteria Isolated from a Waste Sorting Plant Located in Poland―A Case Study

**DOI:** 10.3390/microorganisms8020202

**Published:** 2020-01-31

**Authors:** Ewa Brągoszewska, Izabela Biedroń, Wojciech Hryb

**Affiliations:** 1 Faculty of Power and Environmental Engineering, Department of Technologies and Installations for Waste Management, Silesian University of Technology, 18 Konarskiego St., 44-100 Gliwice, Poland; wojciech.hryb@polsl.pl; 2 Institute for Ecology of Industrial Areas, Environmental Microbiology Unit, 6 Kossutha St., 40-844 Katowice, Poland; izabiedron@gmail.com

**Keywords:** air quality, bacterial aerosol (BA), bioaerosol, antibiotic resistance, waste sorting plant

## Abstract

International interests in biological air pollutants have increased rapidly to broaden the pool of knowledge on their identification and health impacts (e.g., infectious, respiratory diseases and allergies). Antibiotic resistance and its wider implications present us with a growing healthcare crisis, and an increased understanding of antibiotic-resistant bacteria populations should enable better interpretation of bioaerosol exposure found in the air. Waste sorting plant (WSP) activities are a source of occupational bacterial exposures that are associated with many health disorders. The objectives of this study were (a) to assess bacterial air quality (BAQ) in two cabins of a WSP: preliminary manual sorting cabin (PSP) and purification manual sorting cabin (quality control) (QCSP), (b) determine the particle size distribution (PSD) of bacterial aerosol (BA) in PSP, QCSP, and in the outdoor air (OUT), and (c) determine the antibiotic resistance of isolated strains of bacteria. Bacterial strains were identified on a Biolog GEN III (Biolog, Hayward, CA, USA), and disc diffusion method for antimicrobial susceptibility testing was carried out according to the Kirby–Bauer Disk Diffusion Susceptibility Test Protocol. A large share of fecal bacteria, *Enterococcus faecalis* and *Alcaligenes faecalis* spp. *feacalis,* was found in the tested indoor air, which is a potential health hazard to the workers of the monitored WSP. Our results demonstrate the necessity to take into account fecal air pollution levels to avoid making erroneous assumptions regarding the environmental selection of antibiotic resistance. Total elimination of many anthropogenic sources is not possible, but important findings of this study can be used to develop realistic management policies methods to improve BAQ.

## 1. Introduction

Bioaerosols are crucial indicators of air pollution and play an instrumental role as risk factors when it comes to the adverse health outcome [1]. These indicators, also known as primary biological airborne particles (PBAPs), have been linked to various health effects, from allergic, through infections, to toxic reactions [2,3,4,5,6]. PBAPs include all particles having a biological source that is in suspension in the air (bacteria, fungi, viruses, pollen) as well as biomolecules (toxins, debris from membranes such as lipids and proteins) [7].

Bacterial air quality (BAQ) is an important problem because people inhale nearly 10 L of air a minute, which amounts to 15,000 L/day [8]. Waste sorting plants (WSPs) are a specific source of bacteria emission into the air. A WSP occurs during waste transport and processing at sorting stations. Rapid population growth and urbanization around the world has led to increased waste generation rates. In Poland and in many other countries, there are still no established legal limits for occupational exposure to bacteria aerosol in WSPs. However, this information is indispensable for the assessment of population exposure, as well as for the identification of the sources of bacterial aerosols (BAs) emission [9]. 

In the European Union, the protection of workers against hazards related to exposure to biological agents is regulated by Directive 2000/54/EC [10]. Additionally, the harmfulness of these factors in Polish regulations is set out in the regulation dated 22 April 2005 on harmful biological factors for health in the work environment and health protection of employees exposed to these factors [11]. 

Growing concern over the threat posed by antibiotic-resistant bacteria present in the air has turned attention also to the environmental dimensions of the problem, and the receiving environments form another possible hotspot for antibiotic resistance dissemination when bacteria originating from a WSP come in contact with environmental bacteria [12]. The genes that make up this environmental resistome have the potential to be transferred to pathogens, and indeed there is some evidence that at least some clinically relevant resistance genes have originated in environmental microbes [13]. Over the past years, the role of the environment as an important source and dissemination route of resistance has been increasingly recognized [13,14,15], but our knowledge of its contribution is still limited.

This paper aims (a) to asses bacterial air quality (BAQ) in two cabins of WSP: preliminary manual sorting cabin (PSP) and purification manual sorting cabin (quality control) (QCSP), (b) determine concentration and bacterial particle size distribution (PSD) in PSP, QCSP, and in the outdoor air (OUT), and (c) determine the antibiotic resistance of isolated strains of bacteria.

## 2. Experiments 

### 2.1. Sampling Sites

**Figure 1:**
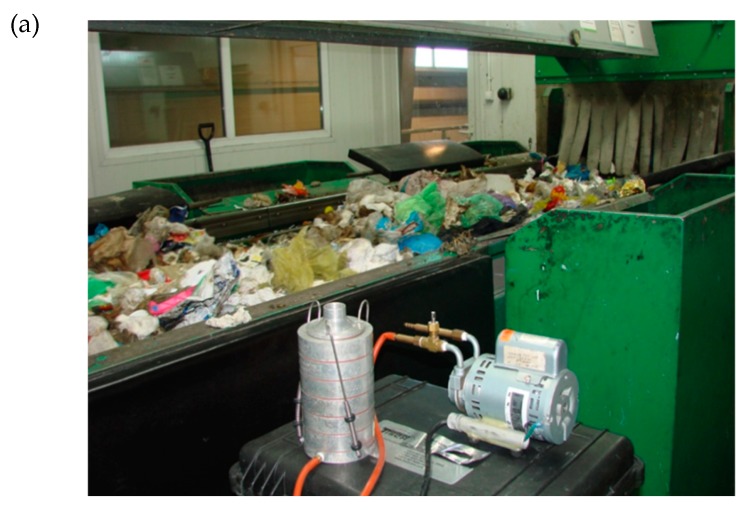

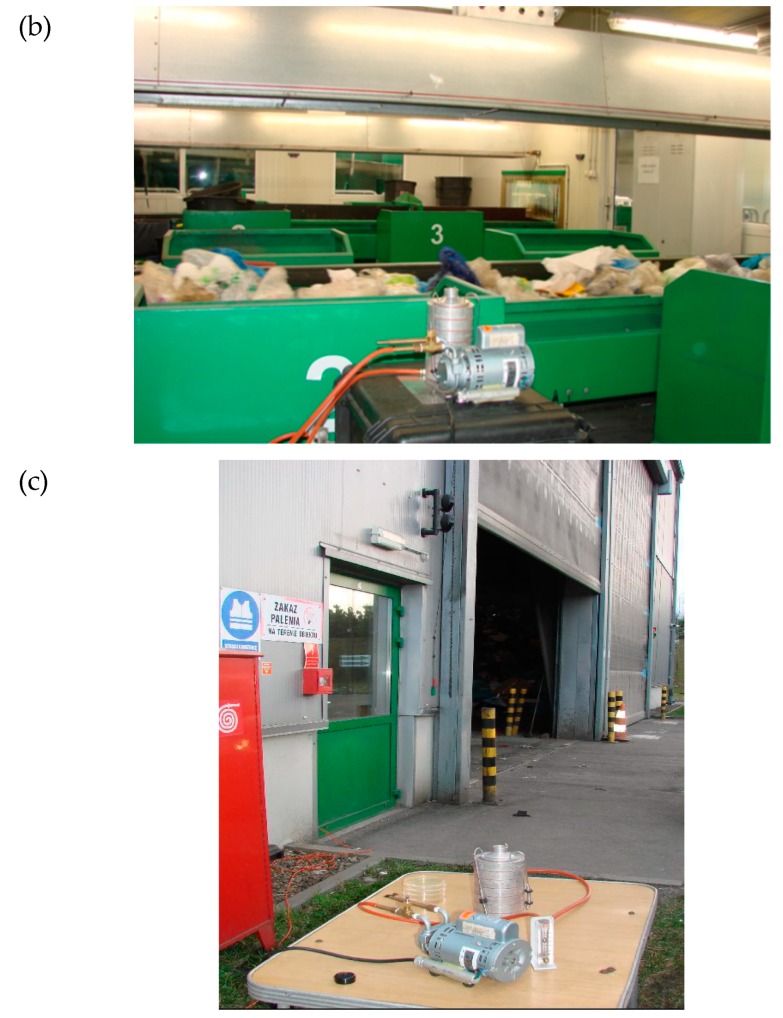
Six-stage Andersen Cascade Impactor (ACI) used during measurements in (**a**) preliminary manual sorting cabin (PSP), (**b**) a purification manual sorting cabin (quality control) (QCSP), and (**c**) outside the analyzed building (OUT).

The study was carried out in two cabins of a WSP: PSP and QCSP, for mixed municipal waste, as well as outside (OUT) the building (Figure 1). The research was conducted during March 2019. Every measurement was conducted between 12:00 am and 15:00 am, when the indoor temperature was about 17 °C, and outdoor was an average of 12 °C. Relative humidity of indoor air (RH) was about 20%, and outdoor 28%. The device used for air temperature and humidity measurement was Oregon Scientific™ (WMR200).

The WSP, which has a capacity of 70,000 Mg/year, works in a two-shift system and is equipped with technology adapted to segregating municipal waste, collected selectively. The volume of the preliminary cabin of sorting plant (PSP) is ~178 m^3^, and the volume of cleaning cabin of sorting plant (QCSP) is ~565 m^3^. The preliminary and purification cabin of the sorting plant has a 20-fold air exchange per hour. It is supply and exhaust ventilation, and the air in the cabins is drawn off from the conveyor belts. In each sorting cabin, there average 10 people working.

### 2.2. Sampling and Analytical Methods

Bacteria were collected using a six-stage Andersen Cascade Impactor (ACI) (Thermo Fisher Scientific, Waltham, MA, USA) with cut-off diameters of 7.0, 4.7, 3.3, 2.1, 1.1, and 0.65 μm. The pump ensured a constant flow rate (28.3 dm³/min) throughout the ACI. The sampling time was 10 min, following Nevalainen et al. [16]. The air sampling device was set at a height of 1.5 m. The ACI was disinfected by 70% ethanol-immersed cotton balls between each sampling. Samples were collected on nutrient media in Petri-dishes located on all ACI stages. Tryptic soy agar (TSA, BioMaxima) was used for bacteria, with cycloheximide added to inhibit fungal growth. The concentration of cycloheximide (95%, ACROS Organics™) in culture medium was 500 mg/L. The Petri dishes were incubated for 48 h at 36 ± 1 °C.

### 2.3. Bacteria Identification and Multi-Antibiotic Resistance (MAR) Test

Bacteria identification and multi-antibiotic resistance (MAR) were practiced by using the same operation details as in our previous studies [17,18,19]. Selected strains were identified using the Biolog OmniLog system (Biolog, Haward, CA, USA) and GEN III MicroPlate™. Cultivated bacteria were also tested for the MAR. Thirty-six different antibiotics and their concentrations were chosen to take into consideration the most common species in the literature concerning antibiotic resistance. 

## 3. Results and Discussion

### 3.1. Quantity of Bacterial Aerosol (BA) of Two Cabins of Sorting Plant and Outdoor Air

Table 1 shows the quantity of BA concentration in the indoor and outdoor air of the sorting plant areas. The mean value of the average concentration of the BA was the highest in the PSP, ranging from 1.49 × 10^3^ to 2.7 × 10^3^ CFU/m^3^, while the average concentration in the QCSP ranged from 8.6 × 10^²^ to 1.9 × 10^3^ CFU/m^3^. The outdoor concentration of BA ranged from 6.9 × 10^2^ to 1.7 × 10^3^ CFU/m^3^. 

BA contamination levels on both PSP and QCSP were lower than the threshold values of occupational exposure specified by the Polish Committee for the Highest Permissible Concentrations and Intensities of Noxious Agents in the Workplace (1.0 × 10^5^ CFU/m^3^) [20]. Similar studies carried out in a sorting plant in Finland showed that the maximum value of BA in the WSP ranged from ~500 to ~1500 CFU/m^3^ [21]. The significantly higher average value of BA was recorded in a WSP located in Korea (1.9 × 10^5^ CFU/m^3^) [22].

### 3.2. Particle Size Distribution (PSD) of Bacterial Aerosol (BA) in Two Cabins of Sorting Plant (PSP; QCSP) and Outdoor Air

Table 2 presents the analysis of the average concentration of BA collected from the different stages of ACI in the indoor and outdoor air of the sorting plant areas.

The highest average concentration of BA in the outdoor air observed on the stage with aerodynamic diameter ranging from 3.3 to 4.7 µm. Stages with aerodynamic diameter ranging from 0.65 to 2.1 had the highest concentration of BA of indoor samples, both in QCSP and in PSP. The results suggest the existence of potential exposure of workers to respirable particles (less than 3.3 µm) that can reach the trachea, bronchi, and alveoli, contributing to adverse respiratory symptoms [23,24].

The indoor/outdoor ratio (I/O) shows us where the source of BA might be found [9,25]. The average I/O calculated for all indoor and outdoor BA total average concentrations was higher than 1, therefore, it could be clearly concluded that the major sources of bioaerosols were internal sources (Table 1). In this case, the major source of the BA is stored waste (especially for fractions <2.1 µm) (Table 2). This result indicates also that there is an additional source of bacterial aerosol for workers of the WSP.

### 3.3. Quality and Antibiotic Resistance of Bacterial Aerosol (BA) in Two Cabins of Sorting Plant (PSP; QCSP)

In a preliminary manual sorting cabin (PSP), a significant dominance of Gram-positive microorganisms (90.48%) was noted, and Gram-positive microorganisms (96.21%) predominated also in the purification manual sorting cabin (quality control) (QCSP). Gram-negative microorganisms constituted 9.52% and 3.79%, respectively. Comparing the qualitative composition between microorganisms isolated from air samples in the two cabins, the dominance of the following species was noted: in the PSP, the dominant species were *Staphylococcus saprophyticus*, *Enterococcus faecalis,* and *Alcaligenes faecalis* spp. *feacalis,* while in QCSP, *Mycobacterium setense* and *Micrococcus luteus* were dominant. According to Directive 2000/54/ EC [10] and the Classification of Harmful Biological Factors developed by the Institute of Rural Health in Lublin, Poland [26], the species selected for testing belong to Risk Group I and they are not hazardous for humans in the work environment, however, their long-term inhalation may cause adverse health effects, especially in workers sensitive to this type of air pollution.

In PSP, the dominant species showed resistance from 45.9% (*Enterecoccus faecalis*) to 56.8% (*Staphylococcus saprophyticus* and *Alcaligenes faecalis* spp*. feacalis*) of all tested antibiotics. Bacteria from the *Staphylococcus* genus are present on the skin and mucous membranes. However, despite its universality in the human environment, it can cause numerous diseases [27]. According to the literature, *Staphylococcus saprophyticus* is a Gram-positive coccus and it is associated primarily with urinary tract infections (UTIs), especially among women [28]. According to the literature, it is a strain that shows resistance to most drugs used in this UTI treatment [29,30,31]. The strain isolated in our study from the air sample showed resistance to Ciprofloxacin, Trimethoprim/ Sulfamethoxazole, Nalidixic acid, Ampicillin, Erythromycin, Vancomycin, and Norfloxacin, which are used to treat UTIs. In contrast, the strain was sensitive to the antibiotic nitrofurantoin [28,32]. It seems interesting that the strain is sensitive to the drug present in use since 1952 (Nitrofurantoin) [33] while it shows resistance to the above-mentioned antibiotics later introduced into medical use (Table 3).

*S. saprophyticus* can be differentiated from another coagulase-negative staphylococcus by its resistance to Novobiocin. Like other uropathogens, *S. saprophyticus* utilizes urease to produce ammonia. However, unlike many of these organisms, it cannot reduce nitrate [34]. 

A strain with a very similar resistance pattern *to Staphylococcus saprophyticus* is *Alcaligenes faecalis* spp*. feacalis.* In our research, the resistance pattern of this strain differs from *Staphylococcus saprophyticus* only by its sensitivity to Amoxycillin and Piperacillin and by resistance to Cefepime. This bacterium can be found in the human digestive tract, but if it lowers immunity it can cause disease. *A. faecalis* has been reported in the case of ocular or urinary tract infections [65]. *A. faecalis* also appears in the feces of birds [66]. There have also been cases of isolating this microorganism from water samples [67]. 

*Enterococcus faecalis* is a Gram-positive bacterium that can cause a variety of nosocomial infections, of which UTIs are the most common. These infections can be exceptionally difficult to treat because of drug resistance of many *E. faecalis* isolates [68]. Enterococci are characterized by natural resistance to numerous antibiotics (among them cephalosporins) and also by easy acquired resistance to antibiotics. Infections caused by multiresistant strains are difficult in treatment, and chronic, recurrent, and sometimes fatal infections are described. Enterococcal infections are caused often by *E. faecalis*, rarely by *E. faecium* [69]. In our study, numerous isolates of this strain were isolated in the PSP, but they showed lower resistance to antibiotics compared with previously described strains.

Our attention was drawn to the high proportion of microorganisms associated with urinary tract infections (UTIs) in bioaerosol. The specificity of sorted waste indicates a large share of hygienic waste from households that can be a source of these microorganisms.

In the QCSP, Gram-positive microorganisms predominated with few isolates of Gram-negative microorganisms (a total of 12 colony-forming units). In the isolates of the two predominant species, *Micrococcus luteus* and *Mycobacterium setense*, resistance to 32.43% and 27.03% of the tested antibiotics was also noted, respectively.

*Mycobacterium setense*, which belongs to nontuberculosis mycobacteria (NTM), is an organism that is increasingly isolated in humans. However, there are also reports of environmental isolates, however, these were samples from hospital spaces [70,71]. This work shows that it is a strain that is no longer only characteristic for the hospital environment. It has already been proven that this microorganism is also able to adapt to other environments, as well as acquire resistance to various disinfectants. *M. setense* might represent an health hazard because, as seen in literature, it is occasionally responsible for opportunistic infections [72,73].

## 4. Conclusions

The research of the bacterial air quality (BAQ) was carried out in two cabins of a waste sorting plant (WSP): preliminary manual sorting cabin (PSP) and purification manual sorting cabin (quality control) (QCSP), as well as outside (OUT) the building. 

The obtained results of particle size distribution (PSD) of bacterial aerosol (BA) may indicate that BA particles come directly from sorted waste. The results suggest the existence of potential exposure of workers to respirable particles (<3.3 µm) that can reach the trachea, bronchi, and alveoli, contributing to adverse respiratory symptoms.

Of the airborne bacteria isolated from air samples in the two cabins, the dominance of the following species was noted: in the PSP, the dominant species were *Staphylococcus saprophyticus*, *Enterecoccus faecalis*, and *Alcaligenes faecalis* spp*. feacalis,* while in QCSP, *Mycobacterium setense* and *Micrococcus luteus* were dominant.

The high proportion of bacteria associated with urinary tract infections (UTIs) was observed. The specificity of sorted waste indicates a large share of hygienic waste from households that can be a source of these bacteria. Additionally, our results demonstrate the obligation to take into account fecal air pollution levels to avoid making erroneous assumptions regarding the environmental selection of antibiotic resistance.

The diversity of resistance genes (RGs) present in the environment suggests that there are still many more resistance genes available for pathogens to recruit. These genes are common among the bacterial populations in the human microbiome and are not likely to be eradicated, even in the absence of antibiotic selection [74]. Therefore, it is important to use personal protective equipment (respiratory protection masks, footwear, protective clothing, and gloves), effective and efficient ventilation, as well as limiting the employees’ working time in these conditions [75]. Although, there is a lack of BAQ standards in Polish legislation, the key problem is keeping a high standard of air quality, and we hope that the results of this campaign may indicate the usefulness of periodic microbiological environmental monitoring to verify the quality of the air and to establish possible technologically achievable guide levels of contamination for a specific work environment. 

## Figures and Tables

**Table 1:** Average concentration and indoor/outdoor ratio (I/O), CFU/m^3^ of total bacterial colony-forming units per cubic meter of preliminary manual sorting cabin (PSP), purification manual sorting cabin (quality control) (QCSP), and outside the analyzed building (OUT).

	PSP	QCSP	OUT
Total average concentration	1.81 × 10³	1.25 × 10³	1.14 × 10³
Min value	1.49 × 10³	8.6 × 10²	6.9 × 10²
Max value	2.7 × 10³	1.9 × 10³	1.7 × 10³
Indoor/outdoor ratio	1.6	1.1	-
SD	8.0 × 10²	6.2 × 10²	5.1 × 10²

**Table 2:** Average concentration and indoor/outdoor ratio (I/O), CFU/m^3^ of bacterial colony-forming units per cubic meter collected from the different stages of ACI in the preliminary manual sorting cabin (PSP), purification manual sorting cabin (quality control) (QCSP), and outside the analyzed building (OUT).

	PSP	QCSP	OUT	I/O for PSP	I/O for QCSP
0.65–1.1	6.36 × 10²	3.26 × 10²	1.59 × 10²	4.0	2.1
>1.1–2.1	6.17 × 10²	3.76 × 10²	2.05 × 10²	3.0	1.8
>2.1–3.3	3.45 × 10²	2.14 × 10²	2.62 × 10²	1.3	0.8
>3.3–4.7	0.55 × 10²	1.63 × 10²	3.19 × 10²	0.2	0.5
>4.7–7.0	1.09 × 10²	1.0 × 10²	1.37 × 10²	0.8	0.7
> 7.0	0.55 × 10²	0.75 × 10²	0.57 × 10²	1.0	1.3

**Table 3:** The pattern of antibiotic resistance of the isolated strains, including the date of introduction of antibiotics for medical use. Marked boxes indicate resistance.

Approved for Medical Use	Antibiotic	*Staphylococcus saprophyticus*	*Enterecoccus faecalis*	*Alcaligenes faecalis* spp. *feacalis*	*Micrococcus luteus E.*	*Mycobacterium setense*
1944	Neomycin [33]					
1952	Erythromycin [35]					
1952	Nitrofurantoin [36]					
1956	Vancomycin [37]					
1960	Metronidazole [38]					
1961	Novobiocin [39,40]					
1962	Trimethoprim [41]					
1963	Ampicillin [42]					
1963	Gentamicin [33]					
1967	Doxycycline [43]					
1967	Nalidixic acid [44]					
1968	Rifampicin [45]					
1968	Tobramycin [33]					
1968	Trimethoprim/sulph [41]					
1970	Ticarcillin [46]					
1971	Minocycline [47]					
1971	Mupirocin [48,49]					
1972	Amoxycillin [42]					
1975	Netilmicin [33]					
1976	Amikacinv [33]					
1977	Cefoxitin [50]					
1978	Cefadroxil [51]					
1979	Cefaclor [42]					
1981	Piperacillin [52,53]					
1983	Norfloxacinv [54,55]					
1985	Ceftazidime [42]					
1985	Ofloxacin [53]					
1985	Aztreonam [56]					
1986	Ciprofloxacin [53]					
1987	Imipenem [57]					
1988	Teicolpanin [58,59]					
1991	Azithromycin [60,61]					
1996	Cefepime [42]					
2001	Ertapenem [62]					
2005	Doripenem [63]					
2010	Ceftaroline [64]					

## References

[1] Jiayu C., Qiaoqiao R., Feilong C., Chen L., Jiguo W., Zhendong W., Lingyun C., Liu R., Guoxia Z. (2019). Microbiology Community Structure in Bioaerosols and the Respiratory Diseases. J. Environ. Sci. Public Health.

[2] Pearson C., Littlewood E., Douglas P., Robertson S., Gant T.W., Hansell A.L. (2015). Exposures and health outcomes in relation to bioaerosol emissions from composting facilities: A systematic review of occupational and community studies. J. Toxicol. Environ. Health. Part. B Crit. Rev..

[3] Fung F., Hughson W.G. (2003). Health effects of indoor fungal bioaerosol exposure. Appl. Occup. Environ. Hyg..

[4] Douwes J., Thorne P., Pearce N., Heederik D. (2003). Bioaerosol health effects and exposure assessment: Progress and prospects. Ann. Occup. Hyg..

[5] Kim K.H., Kabir E., Jahan S.A. (2018). Airborne bioaerosols and their impact on human health. J. Environ. Sci..

[6] Górny R.L. (2020). Microbial Aerosols: Sources, Properties, Health Effects, Exposure Assessment—A Review. KONA Powder Part. J..

[7] Wéry N. (2014). Bioaerosols from composting facilities-a review. Front. Cell. Infect. Microbiol..

[8] Wood R.A., Burchett M.D., Orwell R.A., Tarran J., Torpy F. (2002). Plant/soil capacities to remove harmful substances from polluted indoor air. J. Horticul. Sci. Biotechnol..

[9] Brągoszewska E., Mainka A., Pastuszka J., Lizończyk K., Desta Y. (2018). Assessment of Bacterial Aerosol in a Preschool, Primary School and High School in Poland. Atmosphere.

[10] (2000). Directive 2000/54/EC of the European Parliament and of the Council of 18 September 2000 on the protection of workers from risks related to exposure to biological agents at work. Off. J. Eur. Commun..

[11] Regulation of the Minister of Health dated April 22, 2005 (Journal of Laws of 2005 No. 81, item 716, as amended and Journal of Laws 2008, No. 48, item 288), in Polish. http://prawo.sejm.gov.pl/isap.nsf/download.xsp/WDU20050810716/O/D20050716.pdf.

[12] Karkman A., Pärnänen K., Larsson D.G.J. (2019). Fecal pollution can explain antibiotic resistance gene abundances in anthropogenically impacted environments. Nat. Commun..

[13] Wright G.D. (2010). Antibiotic resistance in the environment: A link to the clinic?. Curr. Opin. Microbiol..

[14] Martinez J.L. (2009). The role of natural environments in the evolution of resistance traits in pathogenic bacteria. Proc. Biol. Sci..

[15] McKinney C.W., Pruden A. (2012). Ultraviolet disinfection of antibiotic resistant bacteria and their antibiotic resistance genes in water and wastewater. Environ. Sci. Technol..

[16] Nevalainen A., Pastuszka J., Liebhaber F., Willeke K. (1992). Performance of bioaerosol samplers: Collection characteristics and sampler design considerations. Atmos. Environ. Part A Gen. Top..

[17] Brągoszewska E., Biedroń I., Hryb W. (2019). Air Quality and Potential Health Risk Impacts of Exposure to Bacterial Aerosol in a Waste Sorting Plant Located in the Mountain Region of Southern Poland, Around Which There Are Numerous Rural Areas. Atmosphere.

[18] Brągoszewska E., Biedroń I., Kozielska B., Pastuszka J.S. (2018). Microbiological indoor air quality in an office building in Gliwice, Poland: Analysis of the case study. Air Qual. Atmos. Health.

[19] Bragoszewska E., Biedroń I. (2018). Indoor air quality and potential health risk impacts of exposure to antibiotic resistant bacteria in an office rooms in southern poland. Int. J. Environ. Res. Public Health.

[20] Skowroń J., Górny R., Augustyńska D., Pośniak M. (2012). Harmful biological agents. The Interdepartmental Commission for Maximum Admissible Concentrations and Intensities for Agents Harmful to Health in the Working Environment: Limit Values.

[21] Lehtinen J., Tolvanen O., Nivukoski U., Veijanen A., Hänninen K. (2013). Occupational hygiene in terms of volatile organic compounds (VOCs) and bioaerosols at two solid waste management plants in Finland. Waste Manag..

[22] Park D.U., Ryu S.H., Kim S.B., Yoon C.S. An Assessment of Dust, Endotoxin, and Microorganism Exposure during Waste Collection and Sorting. J. Air Waste Manag. Assoc..

[23] Owen M.K., Ensor D.S., Sparks L.E. (1992). Airborne particle sizes and sources found in indoor air. Atmos. Environ. Part A Gen. Top..

[24] Lacey J., Dutkiewicz J. (1994). Bioaerosols and occupational lung disease. J. Aerosol Sci..

[25] Faridi S., Hassanvand M.S., Naddafi K., Yunesian M., Nabizadeh R., Sowlat M.H., Kashani H., Gholampour A., Niazi S., Zare A. (2015). Indoor/outdoor relationships of bioaerosol concentrations in a retirement home and a school dormitory. Environ. Sci. Pollut. Res..

[26] Dutkiewicz J., Śpiewak R., Jabłoński L., Szymańska J. (2007). Biological Occupational Risk Factors. Classification, Exposed Occupational Groups, Measurement, Prevention.

[27] Heo Y., Park J., Lim S.I., Hur H.G., Kim D., Park K. (2010). Size-resolved culturable airborne bacteria sampled in rice field, sanitary landfill, and waste incineration sites. J. Environ. Monit..

[28] Ebrahimi K., Alipour M., Yahyapour Y. (2018). Evaluation of antibiotic resistance pattern in *Staphylococcus saprophyticus* isolated from patients with urinary tract infection using real-time PCR. Int. J. Mol. Clin. Microbiol..

[29] Raz R., Colodner R., Kunin C.M. (2005). Who Are You-Staphylococcus saprophyticus?. Clin. Infect. Dis..

[30] Kuroda M., Yamashita A., Hirakawa H., Kumano M., Morikawa K., Higashide M., Maruyama A., Inose Y., Matoba K., Toh H. (2005). Whole genome sequence of *Staphylococcus saprophyticus* reveals the pathogenesis of uncomplicated urinary tract infection. Proc. Natl. Acad. Sci. U S A.

[31] Flores-Mireles A.L., Walker J.N., Caparon M., Hultgren S.J. (2015). Urinary tract infections: Epidemiology, mechanisms of infection and treatment options. Nat. Rev. Microbiol..

[32] Martins K.B., Ferreira A.M., Pereira V.C., Pinheiro L., de Oliveira A., de Lourdes Ribeiro de Souza da Cunha M. (2019). In vitro Effects of Antimicrobial Agents on Planktonic and Biofilm Forms of Staphylococcus saprophyticus Isolated From Patients With Urinary Tract Infections. Front. Microbiol..

[33] Oliveira J.F.P., Cipullo J.P., Burdmann E.A. (2006). Nefrotoxicidade dos aminoglicosídeos. Brazilian Journal of Cardiovascular Surgery.

[34] Ehlers S., Merrill S.A. (2018). Staphylococcus saprophyticus.

[35] Flynn E.H., Sigal M.V., Wiley P.F., Gerzon K. (1954). Erythromycin. I. Properties and Degradation Studies. J. Am. Chem. Soc..

[36] Waisbren B.A., Crowley W. (1955). Nitrofurantoin: Clinical and laboratory evaluation. A.M.A. Arch. Intern. Med..

[37] Levine D.P. (2006). Vancomycin: A History. Clin. Infect. Dis..

[38] Roe F.J.C. (1977). Metronidazole: View of uses and toxicity. J. Antimicrob. Chemother..

[39] Fairbrother R.W., Williams B.L. (1956). Two new antibiotics. Lancet.

[40] Bisacchi G.S., Manchester J.I. (2015). A New-Class Antibacterial-Almost. Lessons in Drug Discovery and Development: A Critical Analysis of More than 50 Years of Effort toward ATPase Inhibitors of DNA Gyrase and Topoisomerase IV. ACS Infect. Diseases.

[41] Eliopoulos G.M., Huovinen P. (2001). Resistance to Trimethoprim-Sulfamethoxazole. Clin. Infect. Dis..

[42] Mira P.M., Crona K., Greene D., Meza J.C., Sturmfels B., Barlow M. (2015). Rational design of antibiotic treatment plans: A treatment strategy for managing evolution and reversing resistance. PLoS ONE.

[43] Li J.J., Li J.J., Corey E.J. (2013). History of drug discovery. Drug Discovery: Practices, Processes, and Perspectives.

[44] Emmerson A.M. (2003). The quinolones: Decades of development and use. J. Antimicrob. Chemother..

[45] Sensi P. (1983). History of the development of rifampin. Rev. Infect. Dis..

[46] Neu H.C., Winshell E.B. (1971). Semisynthetic Penicillin 6-[d(—)-α-Carboxy-3-Thienylacetamido] Penicillanic Acid Active Against Pseudomonas In Vitro. Appl. Environ. Microbiol..

[47] Brogden R.N., Avery G.S. (1972). New Antibiotics: Epicillin, Minocycline and Spectinomycin A summary of their antibacterial activity, pharmacokinetic properties and therapeutic efficacy. Drugs.

[48] Fuller A.T., Mellows G., Woolford M., Banks G.T., Barrow K.D., Chain E.B. (1971). Pseudomonic acid: An antibiotic produced by *Pseudomonas fluorescens*. Nature.

[49] Sutherland R., Boon R.J., Griffin K.E., Masters P.J., Slocombe B., White A.R. (1985). Antibacterial activity of mupirocin (pseudomonic acid), a new antibiotic for topical use. Antimicrob. Agents Chemother..

[50] Geddes A.M., Schnurr L.P., Ball A.P., Mcghie D., Brookes G.R., Wise R. (1977). Cefoxitin: A hospital study. Brit. Med. J..

[51] Buck R.E., Price K.E. (1977). Cefadroxil, a new broad spectrum cephalosporin. Antimicrob. Agents Chemother..

[52] Winston D.J., Murphy W., Young L.S., Hewitt W.L. (1980). Piperacillin therapy for serious bacterial infections. Am. J. Med..

[53] Fischer J., Robin Ganellin C. (2006). Analogue-based Drug Discovery.

[54] Ito A., Hirai K., Inque M. (1980). In vitro antibacterial activity of AM-715, a new nalidixic acid analog. Antimicrob. Agents Chemother..

[55] Leigh D.A., Emmanuel F.X.S. (1984). The treatment of *Pseudomonas aeruginosa* urinary tract infections with norfloxacin. J. Antimicrob. Chemother..

[56] Childs S.J. (1985). Aztreonam in the treatment of urinary tract infection. Am. J. Med..

[57] Zhanel G.G., Simor A.E., Vercaigne L., Mandell L. (1998). Imipenem and meropenem: Comparison of in vitro activity, pharmacokinetics, clinical trials and adverse effects. Can. J. Infect. Dis..

[58] Parenti F., Beretta G., Berti M., Arioli V. (1978). Teichomycins, New Antibiotics from Actinoplanes Teichomyceticus Nov. SP. I. Description of the Producer Strain, Fermentation Studies and Biological Properties. J. Antibiot..

[59] Pryka R.D., Rodvold K.A., Rotschafer J.C. (1988). Teicoplanin: An investigational glycopeptide antibiotic. Clin. Pharm..

[60] Amrol D. (2007). Single-dose azithromycin microsphere formulation: A novel delivery system for antibiotics. Int. J. Nanomed..

[61] Whitman M.S., Tunkel A.R. (1992). Azithromycin and Clarithromycin: Overview and Comparison with Erythromycin. Infect. Control. Hosp. Epidemiol..

[62] Livermore D.M., Sefton A.M., Scott G.M. (2003). Properties and potential of ertapenem. J. Antimicro. Chem..

[63] Hilas O., Ezzo D.C., Jodlowski T.Z. (2008). Doripenem (doribax), a new carbapenem antibacterial agent. Pharm. Ther..

[64] Lounsbury N., Reeber M.G., Mina G., Chbib C. (2019). A mini-review on ceftaroline in bacteremia patients with methicillin-resistant *Staphylococcus aureus* (MRSA) infections. Antibiotics.

[65] Momtaz F., Ali M.H., Hossain M.N., Foysal M.J., Sumiya M.K., Islam K. (2018). Characterisation of multidrug-resistant Alcaligenes faecalis strain AF1 isolated from patient of RUTIs: A study from Bangladesh. J. Clin. Diagn. Res..

[66] Filipe M., Reimer Å., Matuschek E., Paul M., Pelkonen T., Riesbeck K. (2017). Fluoroquinolone-resistant Alcaligenes faecalis related to chronic suppurative otitis media, Angola. Emerg. Infect. Dis..

[67] Bizet J., Bizet C. (1997). Strains of Alcaligenes faecalis from clinical material. J. Infect..

[68] Kau A.L., Martin S.M., Lyon W., Hayes E., Caparon M.G., Hultgren S.J. (2005). Enterococcus faecalis tropism for the kidneys in the urinary tract of C57BL/6J mice. Infect. Immun..

[69] Rudy M., Nowakowska M., Wiechuła B., Zientara M., Radosz-Komoniewska H. (2004). Antibiotic susceptibility analysis of *Enterococcus* spp. isolated from urine. Przegl Lek.

[70] Azadi D., Dibaj R., Pourchangiz M., Daei-Naser A., Shojaei H. (2014). First report of isolation of *Mycobacterium* canariasense from hospital water supplies. Scand. J. Infect. Dis..

[71] Keikha M. (2018). Case report of isolation of *Mycobacterium* setense from a hospital water supply. Environ. Dis..

[72] Tille P. (2014). Bailey & Scott’s Diagnostic Microbiology.

[73] Azadi D., Shojaei H., Pourchangiz M., Dibaj R., Davarpanah M., Naser A.D. (2016). Species diversity and molecular characterization of nontuberculous mycobacteria in hospital water system of a developing country, Iran. Microb. Pathog..

[74] Bengtsson-Palme J., Kristiansson E., Larsson D.G.J. (2018). Environmental factors influencing the development and spread of antibiotic resistance. FEMS Microbiol. Rev..

[75] Majchrzycka K., Okrasa M., Jachowicz A., Szulc J., Gutarowska B. (2018). Microbial growth on dust-loaded filtering materials used for the protection of respiratory tract as a factor affecting filtration efficiency. Int. J. Environ. Res. Public Health.

